# The Silencing of the *StPAM16-1* Gene Enhanced the Resistance of Potato Plants to the Phytotoxin Thaxtomin A

**DOI:** 10.3390/ijms26031361

**Published:** 2025-02-06

**Authors:** Lu Liu, Shuangwei Song, Ning Liu, Zhiqin Wang, Yonglong Zhao, Naiqin Zhong, Pan Zhao, Haiyun Wang

**Affiliations:** 1 State Key Laboratory of Plant Genomics, Institute of Microbiology, Chinese Academy of Sciences, Beijing 100101, China; liulu202206@163.com (L.L.); song_shuangwei@163.com (S.S.); wangzq1210@163.com (Z.W.); ylzhao97101@163.com (Y.Z.); nqzhong@im.ac.cn (N.Z.); 2 Engineering Laboratory for Advanced Microbial Technology of Agriculture, Chinese Academy of Sciences, Beijing 100101, China; 3 Beijing Vegetable Research Center (BVRC), Beijing Academy of Agriculture and Forestry Science, Beijing 100097, China; liuning@nercv.org; 4 National Engineering Research Center for Vegetables (NERCV), State Key Laboratory of Vegetable Biobreeding, Beijing Academy of Agriculture and Forestry Sciences, Beijing 100097, China; 5 Key Laboratory of Potato Industry Integration and Development Enterprises in Inner Mongolia Autonomous Region, Hulunbuir 021000, China

**Keywords:** *Solanum tuberosum*, *Streptomyces scabiei*, thaxtomin A, StPAM16-1, StCSN5

## Abstract

Potato common scab (CS) caused by *Streptomyces scabiei* is a severe disease that threatens tuber quality and its market value. To date, little is known about the mechanism regulating the resistance of potato to CS. In this study, we identified a presequence translocase-associated motor 16 gene from potato (designated *StPAM16-1*) that is involved in the response to the phytotoxin thaxtomin A (TA) secreted by *S. scabiei*. The StPAM16-1 protein was localized in the mitochondria, and the expression of the gene was upregulated in potato leaves treated with TA. The suppression of *StPAM16-1* in potato led to enhanced resistance to TA and *S. scabiei*. Protein interaction analyses revealed that StPAM16-1 interacted with the subunit 5b of the COP9 signalosome complex (StCSN5). Similar to that of *StPAM16-1*, the expression levels of *StCSN5* significantly increased in potato leaves treated with TA. These results indicated that StPAM16-1 acted as a negative regulator and was functionally associated with StCSN5 in the immune response of potato plants against CS. Our study sheds light on the molecular mechanism by which PAM16 participates in the plant immune response. Furthermore, both *StPAM16-1* and *StCSN5* could be potential target genes in the molecular breeding of potato cultivars with increased resistance to CS.

## 1. Introduction

Potato common scab (CS), caused by pathogenic *Streptomyces* spp., is a severe soil-borne disease worldwide [1]. The symptoms of CS include scab-like superficial, raised and/or pitted lesions at infection sites, which results in a decrease in the quality and marketability of potato tubers [2]. Previous studies revealed that the phytotoxin thaxtomin, which is secreted by pathogenic *Streptomyces* spp., including *S. scabiei*, is the key pathogenic factor of CS [3]. As the predominant pathogenic component of the thaxtomin family, thaxtomin A (TA) affects plant growth and development in various ways, such as by inhibiting plant cellulose biosynthesis [4] and seedling growth [5], causing dramatic cell hypertrophy [6], altering Ca^2+^ and H^+^ flow [7,8] and triggering programmed cell death (PCD) [9]. Although several approaches such as crop rotation, chemical treatment and biological control have been shown to alleviate this disease, control efficiency is limited [10]. Given that disease resistance in potato cultivars is the foundation of the integrated method, studying the genetics of tolerance in breeding is regarded as one of the best options for controlling CS. To date, the strongest source of resistance has been a Phureja group clone, and a major dominant resistance gene is in the populations of hybrids between *Solanum tuberosum* dihaploids and Phureja group clones [11,12]. Research on the genome evolution and diversity of wild and cultivated potatoes, as well as the graph pangenome [13,14,15], can provide critical insights into the potato genome and guide breeders in potato breeding. At present, the identification and functional characterization of defense-related genes to generate CS-resistant cultivars via molecular breeding are some of the desirable strategies for controlling CS.

The presequence translocase-associated protein import motor (PAM) subunit PAM16 is required for protein precursors to be translocated into the mitochondrial matrix [16]. In *Arabidopsis*, AtPAM16 may play a negative role in plant immunity by importing a negative regulator of plant immunity to mitochondria to inhibit the overaccumulation of ROS, and a single *Atpam16* mutant plant exhibited increased resistance to virulent pathogens [17]. *Atpam16-1* and *Atpam16-2* double-mutant plants showed enhanced SUPPRESSOR OF NPR1-1, CONSTITUTIVE 1 (SNC1)-mediated immunity [17]. The mechanism of non-target site resistance to the herbicide thaxtomin A conferred by the gene *pam16* mutant in *Marchantia polymorpha* increases ROS levels or decreases thaxtomin A metabolism in weeds [18]. The homologous gene thaxtomin resistance 1 (*TXR1*) was first identified during the screening of a TA-resistant mutant of *Arabidopsis* [5]. The study revealed that the uptake of TA by *txr1*-transformed plants was lower than that by wild-type (WT) plants, but the metabolism of TA did not differ between the WT and *txr1* mutant plants. These results implied that PAM16 (TXR1) likely plays an important role in plant responses to biotic stresses. In human cells, a protein homologous to yeast PAM16 named Magmas (mitochondria-associated granulocyte-macrophage colony-stimulating factor signaling molecule) was found to be an essential part of the mitochondrial precursor protein import machinery [19]. In addition, it was reported that Magmas can regulate cellular ROS levels by controlling ROS production and scavenging [20]. Therefore, it is of great interest to study how PAM16 regulates the plant immune system as a mitochondrial intima input protein and to analyze its physiological functions and mechanisms, for example, in potato responses to *S. scabiei* infection and TA treatment.

COP9 signalosome (CSN) is a conserved and nucleus-localized protein complex that is fractionated as a 450–550 kDa complex in gel filtration columns [21,22,23]. Studies have shown that CSN regulates the activity of CULLIN-RING E3 ubiquitin ligases (CRLs) by removing the ubiquitin-like protein RUB1 (related to ubiquitin 1 in plants)/NEDD8 (neural precursor cell-expressed developmentally downregulated-8 in animals), which interact with CRLs and thus affect the development and defense against pathogens and herbivorous insects [24]. CSN consists of eight subunits, among which CSN5 is a metalloprotease with a conserved JAMM (JAB1 MPN domain metalloprotease) motif and requires zinc ions as an activator [21,25]. CSN is widely present in eukaryotes. In plants, CSN plays an important role in regulating development and stress responses [26,27,28,29,30] and acts as an inhibitor of photomorphogenesis in *Arabidopsis* [31].

The identification and functional characterization of defense-related genes not only provide information for the interaction between potato and CS but also help in identifying candidate genes for molecular breeding to generate CS-resistant cultivars. In this study, we identified a phytotoxin TA-responsive gene, *StPAM16-1*, from tetraploid potato (*Solanum tuberosum* L.) cv. Shepody and found that the relative expression of *StPAM16-1* was induced by TA treatment and that StPAM16-1 was localized in mitochondria. We characterized the function of StPAM16-1 in response to TA treatment by VIGS and RNAi. StPAM16-1 could interact with StCSN5 via yeast two-hybrid and split luciferase complementation assays. Our results demonstrated that StPAM16-1 acted as a negative regulator of the defense response to TA in potato. As the expression of *StCSN5* was also induced by TA treatment, we speculated that the interaction between StPAM16-1 and StCSN5 may reflect a functional link between the two proteins in the immune response of potato to CS.

## 2. Results

### 2.1. Identification of StPAM16-1

**Figure 1:**
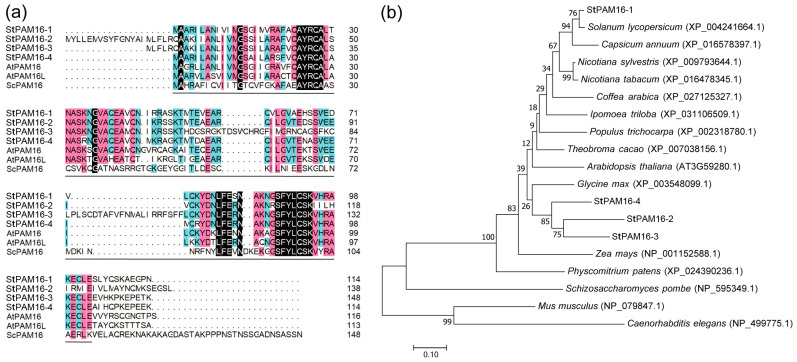
Homology analysis of StPAM16-1. (**a**,**b**) show multiple sequence alignment and phylogenetic analysis of StPAM16-1 and its homologous proteins, respectively. DnaJ domain is underlined by solid lines. Different colors of background represent similarity of amino acid sequences (black: 100%; pink: 75%; blue: 50%). Scale bar stands for evolutionary distance.

A previous study reported that *AtPAM16*/*AtTXR1* is a TA-responsive gene that plays a negative role in the defense process in *Arabidopsis* [5]. In the aim of improving the disease tolerance of potato plants through genetic manipulation, we selected *PAM16* as the candidate gene and identified AtPAM16/AtTXR1 orthologs in the potato proteome via sequence alignment via the Potato Genomics Resource (http://solanaceae.plantbiology.msu.edu/; accessed on 15 November 2021) and NCBI databases (https://blast.ncbi.nlm.nih.gov/; accessed on 15 November 2021). A total of four proteins with high homology to AtPAM16/AtTXR1 were identified, among which the protein with the highest homology was named StPAM16-1 (PGSC0003DMP400044341). The other three homologous proteins were named StPAM16-2 (PGSC0003DMP400051461), StPAM16-3 (PGSC0003DMP400051472) and StPAM16-4 (PGSC0003DMP400026161), respectively. The sequence alignment and domain analysis of StPAM16-1, StPAM16-2, StPAM16-3, StPAM16-4, AtPAM16, AtPAM16L and ScPAM16 were carried out using DNAMAN. StPAM16-1 contains a highly conserved DnaJ domain (Figure 1a), which mediates chaperone binding [32]. Furthermore, the phylogenetic analysis of StPAM16-1 and its homologous proteins was carried out by MEGA 7.0. As shown in Figure 1b, PAM16 is present in various eukaryotes, and StPAM16-1 shares high homology with the PAM16 of *S. lycopersicum*.

### 2.2. StPAM16-1 Expression Is Induced by TA and Defense-Related Plant Hormones

**Figure 2:**
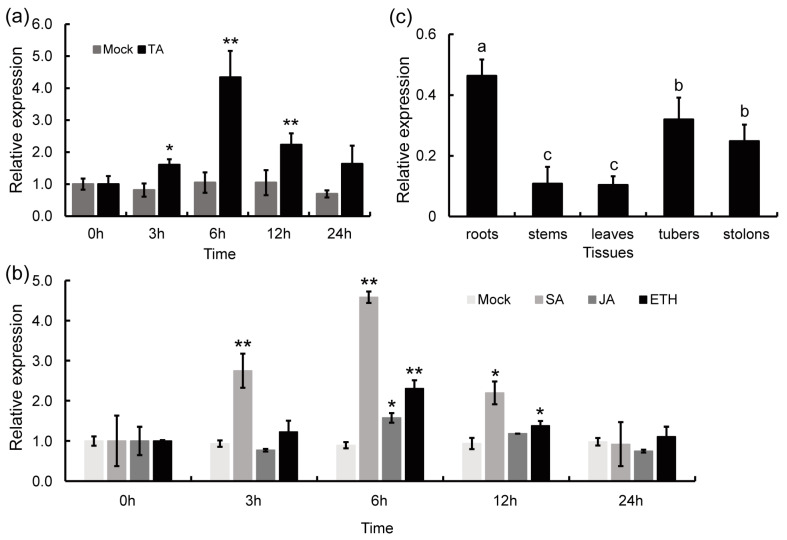
The expression pattern of *StPAM16-1* in potato plants. (**a**,**b**) The relative expression of *StPAM16-1* in potato leaves after TA (**a**), SA, JA and ETH (**b**) treatments. The asterisks denote statistically significant differences according to Student’s *t* test; * *p* < 0.05, ** *p* < 0.01. (**c**) The relative expression of *StPAM16-1* in different organs of potato. Different letters indicate statistically significant differences as determined by Student’s *t* test. *StActin* was used as a reference gene. The values represent the means ± standard deviation (SD). Three biological replicates were performed.

To test whether StPAM16-1 participates in the response to TA in potato, *StPAM16-1* transcript levels were determined by quantitative reverse transcription PCR (RT-qPCR). The results showed that the relative expression of *StPAM16-1* was significantly upregulated at 6 h post-treatment and then gradually recovered to the background level (Figure 2a). Then, we examined the relative expression of *StPAM16-1* after treatment with several defense-related phytohormones, including salicylic acid (SA), jasmonic acid (JA) and ethylene (ETH). The results showed that the expression of *StPAM16-1* was upregulated after treatment with SA, JA and ETH (Figure 2b), with the highest enhancement occurring after SA treatment.

In addition, the relative expression of *StPAM16-1* in different tissues was detected by RT-qPCR. As shown in Figure 2c, *StPAM16-1* was expressed in all tissues but with relatively higher levels in the roots, tubers and stolons, which is closely related to its disease-related function.

### 2.3. StPAM16-1 Localizes to Mitochondria

**Figure 3:**
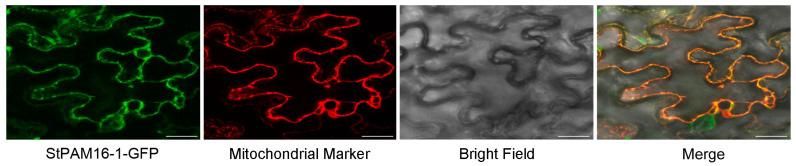
Subcellular localization of StPAM16-1 in *Nicotiana benthamiana* leaf cells. Bar = 25 μm.

To further understand the biological function of StPAM16-1, the subcellular distribution of the proteins was analyzed using a tobacco transient expression system. An StPAM16-1-GFP fusion gene driven by the 35S promoter was constructed in the vector and transformed into competent Agrobacterium cells. The transformants were then injected into tobacco leaf cells. As shown in Figure 3, StPAM16-1-GFP fusion proteins were colocalized with mitochondrial markers, indicating that StPAM16-1 is localized to this organelle.

### 2.4. Suppression of StPAM16-1 Expression Improves Tolerance of Potato Plants to Thaxtomin A and S. scabiei

**Figure 4:**
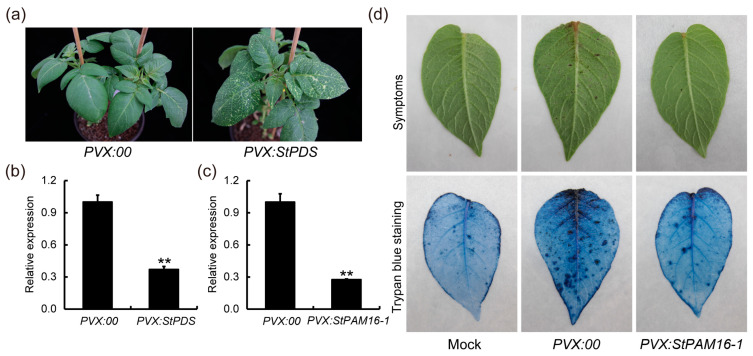
Increased tolerance of *StPAM16-1*-silenced potato plants to TA treatment. (**a**) Albino phenotype of control (*PVX:00*) and *StPDS*-silenced potato plants (*PVX:StPDS*) approximately 40 days after injection. (**b**) Relative expression of *StPDS* in *PVX:00* and *PVX:StPDS*. (**c**) Relative expression of *StPAM16-1* in PVX:00 and *StPAM16-1*-silenced potato plants (*PVX:StPAM16-1*). *StActin* was used as reference gene. Values represent means ± SD. Asterisks denote statistically significant differences as determined by Student’s *t* test, ** *p* < 0.01. Three biological replicates were performed. (**d**) Phenotypes of *PVX:00* and *PVX:StPAM16-1* after TA treatment, which were stained with trypan blue. Mock represents *PVX:00* treated with sterile water.

To investigate the function of StPAM16-1 in response to TA, we first assessed the disease tolerance of potato plants with decreased *StPAM16-1* expression levels via virus-induced gene silencing (VIGS). To test the efficiency of the VIGS system, *StPDS*, which encodes a 15-cis-phytoene desaturase, was used as a marker. Approximately 40 days after injection, the albino phenotype appeared on *StPDS*-silenced potato leaves, and *StPDS* silencing was verified by RT-qPCR, which confirmed the efficiency of VIGS (Figure 4a,b). Then, we constructed the VIGS vector *pgR107-StPAM16-1* and transformed it into *Agrobacterium*. About 40 days after injection, total RNA was extracted from the leaves to determine the relative expression of *StPAM16-1* via RT-qPCR. The results showed that the relative expression of *StPAM16-1* was significantly lower in *StPAM16-1*-silenced potato leaves than in the control (Figure 4c). Subsequently, *StPAM16-1*-silenced and control potato plants were treated with 10 μM TA to test their tolerance. As shown in Figure 4d, there were more black necrotic spots on the control potato leaves than on the *StPAM16-1*-silenced potato leaves. Then, we used trypan blue to stain potato leaves treated with TA or sterile water and found that the necrosis of control potato leaves was more severe than that of *StPAM16-1*-silenced leaves. These results indicated that silencing *StPAM16-1* increased the tolerance of potato plants to TA.

**Figure 5:**
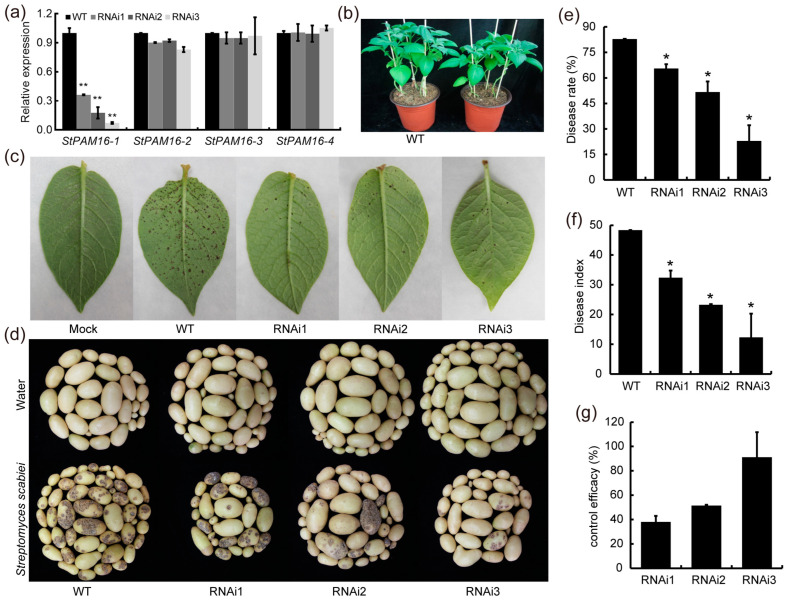
Repression of *StPAM16-1* expression enhanced tolerance of potato plants to TA and *S. scabiei* infection. (**a**) Relative expression levels of *StPAM16-1* and its homologous genes in WT and *StPAM16-1*-RNAi potato plants. (**b**) Phenotypes of WT and *StPAM16-1*-RNAi potato plants. (**c**) Repression of *StPAM16-1* expression increased tolerance of potato plants to TA treatment. (**d**) Phenotypes of WT and *StPAM16-1*-RNAi potato tubers after *S. scabiei* infection. Mock represents WT potato tubers treated with sterile water. (**e**) Rate of diseased tubers of WT and *StPAM16-1*-RNAi potato plants. (**f**) Disease index of WT and *StPAM16-1*-RNAi potato tubers. (**g**) Relative disease control effect of *StPAM16-1*-RNAi in potato tubers. Values represent means ± SD. Asterisks denote statistically significant differences according to Student’s *t* test; * *p* < 0.05, ** *p* < 0.01. Three biological replicates were performed.

To further explore the effects of StPAM16-1 in response to TA, the expression of *StPAM16-1* was suppressed by the RNAi approach, and an in-depth analysis was carried out. The *pANDA35K-StPAM16-1* RNAi vector was constructed and subsequently transformed into potato plants via *Agrobacterium*-mediated transformation. The relative expression of *StPAM16-1* in RNAi potato plants was measured by RT-qPCR. Three RNAi plants with different expression levels of *StPAM16-1* were selected and named RNAi1, RNAi2 and RNAi3. As shown in Figure 5a, the relative expression levels of *StPAM16-1* in the three *StPAM16-1*-RNAi plants were significantly lower than those in the WT plant, while the expression of *StPAM16-2*, *StPAM16-3* and *StPAM16-4* did not change in the WT and *StPAM16-1*-RNAi potato plants. Compared with the WT plants, the *StPAM16-1*-RNAi plants also exhibited a dwarf phenotype (Figure 5b). This finding is consistent with a previous report on *Arabidopsis* [5]. Therefore, *StPAM16-1*-RNAi plants were selected for further studies.

We treated WT and three *StPAM16-1*-RNAi potato leaves with 10 μM TA. As shown in Figure 5c, the tolerance of *StPAM16-1*-RNAi potato leaves to TA was significantly enhanced as compared to the WT control. Further, WT and *StPAM16-1*-RNAi plants grown for 3–4 weeks were inoculated with *S. scabiei*. Potato tubers were harvested after 3 months, and the disease incidence of tubers was counted. The results showed that there were a large number of scab patches on the WT tubers, while there were fewer scab patches on the *StPAM16-1*-RNAi tubers (Figure 5d). Meanwhile, the incidence rate, disease index and relative control effect of the WT and *StPAM16-1*-RNAi tubers were also calculated. Compared with those of the WT tubers, the incidence rate (Figure 5e) and disease index (Figure 5f) of the *StPAM16-1*-RNAi tubers significantly decreased, while the relative control effect clearly increased (Figure 5g). These results indicated that the inhibition of *StPAM16-1* improved the tolerance of potato plants to *S. scabiei*.

### 2.5. StPAM16-1 Interacts with StCSN5

To elucidate the functional mechanism of StPAM16-1, the cDNA of potato leaves treated with TA was used to construct a yeast two-hybrid library. The cDNA was cloned into the yeast expression vector pGADT7 for prey protein screening, and StPAM16-1 cDNA was inserted into the pGBKT7 vector as bait. Four candidate proteins that interact with StPAM16-1 were initially screened from the yeast two-hybrid library (Table 1), including COP9 signalosome complex subunit 5b-like (StCSN5), anthocyanin-3-O-glucosyltransferase 2-like and fructose-diphosphate aldolase-like protein and the uncharacterized protein LOC102596406.

**Figure 6:**
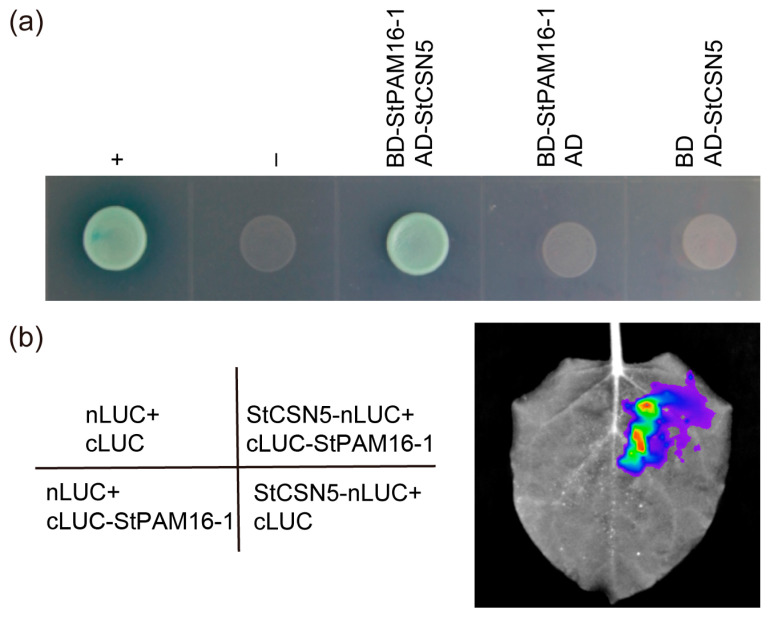
Interaction between StPAM16-1 and StCSN5. (**a**) Interaction between StPAM16-1 and StCSN5 was verified by yeast two-hybrid assay. +, positive control (BD-L53/AD-T); –, negative control (BD-Lam/AD-T). (**b**) Split luciferase complementation assay of interaction between StPAM16-1 and StCSN5 in plant cells.

Among these four candidate proteins, CSN was reported to play an important role in regulating development and stress response [33]. Hence, we selected StCSN5 to verify the interaction between StPAM16-1. Yeast two-hybrid assays revealed a strong interaction between StPAM16-1 and StCSN5 (Figure 6a). Moreover, the interaction between StPAM16-1 and StCSN5 in plant cells was verified by the split luciferase complementation assay. As shown in Figure 6b, when an *Agrobacterium* culture containing cLUC-StPAM16-1/nLUC-StCSN5 vectors was injected into tobacco leaves, strong fluorescence was produced on the leaves. However, no fluorescence was detected in tobacco leaves injected with *Agrobacterium* cultures containing nLUC/cLUC-StPAM16-1, nLUC-StCSN5/cLUC or nLUC/cLUC. The results confirmed that StPAM16-1 and StCSN5 could interact in plant cells.

### 2.6. Expression of StCSN5 Is Significantly Induced by TA and Several Defense-Related Plant Hormones

**Figure 7:**
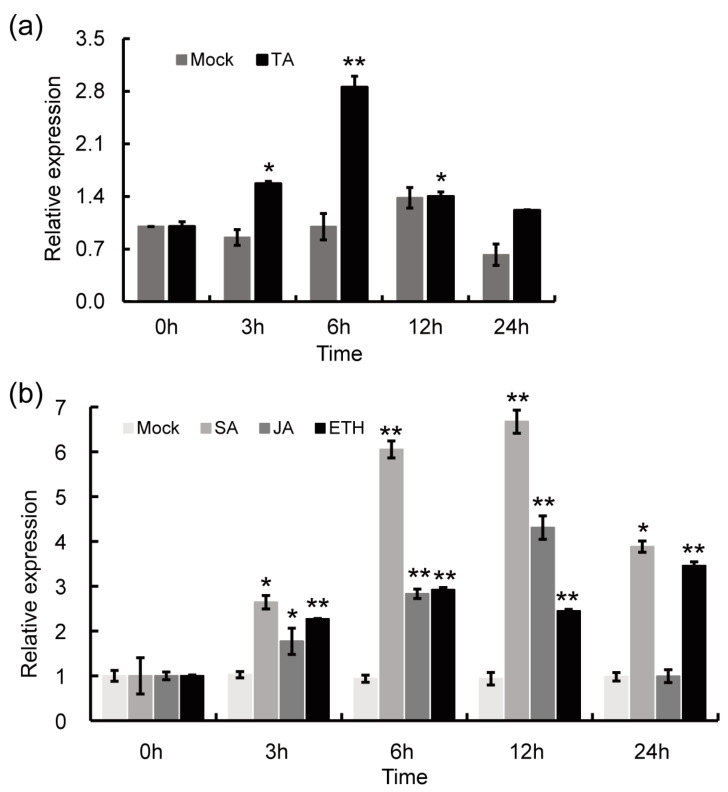
Relative expression of *StCSN5* in potato leaves after TA (**a**), SA, JA and ETH (**b**) treatments. *StActin* was used as reference gene. Values represent means ± SD. Asterisks denote statistically significant differences according to Student’s *t* test; * *p* < 0.05, ** *p* < 0.01. Three biological replicates were performed.

Given that the expression of *StPAM16-1* was induced by TA, we speculated that *StCSN5* expression might also respond to TA treatment. To verify this hypothesis, RT-qPCR was used to analyze the relative expression of *StCSN5* after TA treatment. Figure 6 shows that the transcription of *StCSN5* was indeed induced by TA treatment and peaked at 6 hpi. This expression pattern of *StCSN5* is similar to that of *StPAM16-1*. Hence, it can be speculated that the functions of these two genes are closely related to the responses of potato plants to TA and *S. scabiei* infection (Figure 7a).

Furthermore, we tested the expression of *StCSN5* in potato plants after defense-related hormone treatment by RT-qPCR. The results showed that the expression of *StCSN5* was induced by JA and ETH (Figure 7b) treatments.

## 3. Discussion

As a serious potato disease, CS has been one of the most prevalent concerns for farmers and industry [34]. In our previous study, we found that the phytotoxin TA secreted by pathogenic *S. scabiei* could trigger a series of host immune responses in potato [35], including changes in many metabolic pathways, enzymatic activities and ROS accumulation. Here, we identified a thaxtomin responsive gene from potato, *StPAM16-1*, that negatively regulates potato immune responses against TA and CS.

In this study, through a series of experiments, we showed that the relative expression of *StPAM16-1* in potato leaves was induced by TA (Figure 2a) and that *StPAM16-1*-inhibited potato plants generated by VIGS and RNAi acquired increased resistance to TA and *S. scabiei* (Figure 4 and Figure 5). Plants are constantly confronted with the invasions of a variety of pathogens, including bacteria, fungi, viruses, nematodes and oomycetes [36]. To combat these attacks, plants have an evolved delicate and two-layered innate immune system, i.e., pattern-triggered immunity (PTI) and effector-triggered immunity (ETI) [37]. Many plant proteins participate and play either positive or negative roles in these immune processes in different plant-pathogen interaction systems [38,39]. During the interaction between potato and pathogens, the expression of the *StLysM-RLK05* gene was upregulated in tubers infected with *S. scabiei* [40], and the expression of *StLysM-RLK05* was significantly upregulated in resistant tubers compared with susceptible tubers infected with *S. scabiei*, suggesting a positive regulatory role of the protein in the immune response. In another study, Tai et al. reported that the expression of two *MYB* and three *bHLH* genes was correlated with CS resistance, indicating that these genes may contribute to the defense response of potato against CS [41]. Similarly, in *Arabidopsis thaliana*, AtPAM16/AtTXR1 participated in the response to TA and the pathogens *Pseudomonas syringae* and *Hyaloperonospora arabidopsidis* Noco2 [5,17]. Above all, we believe that StPAM16-1, which is similar to AtPAM16, is a negative regulator of the immune response of potato plants to TA and *S. scabiei* infection.

Similar to the AtPAM16 protein [12], the subcellular distribution analysis revealed that StPAM16-1 is a mitochondria-localized protein (Figure 3). The translocation of mitochondrial preproteins was significantly decreased in the yeast *pam16* mutant compared with the WT [16,42]. Similarly, a subunit of the *Arabidopsis* PAM complex, AtPAM16, was found to bind to the TIM23 complex and participate in translocating mitochondrial preproteins into the matrix [43,44]. Additionally, a previous study reported that TXR1 (PAM16) was a regulator of a transport system, and the increased resistance of the Arabidopsis mutant *txr1* to TA was due to a decrease in the rate of toxin uptake [5]. In our study, we observed that the expression of *StPAM16-1* was significantly induced by TA treatment, suggesting that upregulated *StPAM16-1* might play the same role as AtTXR1 in facilitating the entry of TA toxins into potato cells. Based on this information, we speculated that StPAM16-1 plays a role in response to TA and *S. scabiei* possibly by translocating a preprotein into mitochondria. This preprotein could be TA or a mitochondrial preprotein related to TA. As TA is only a cyclic dipeptide molecule containing tryptophan and phenylalanine residues [45,46], it is likely that TA binds to mitochondrial preproteins and is co-translocated into the mitochondrial matrix. However, such a role of StPAM16-1 in the translocation of mitochondrial preproteins needs to be further assessed.

To investigate the functional mechanism of StPAM16-1, we screened four proteins that interact with StPAM16-1 from a yeast two-hybrid library and verified the interaction between StPAM16-1 and StCSN5 via yeast two-hybrid and split luciferase complementation assays (Figure 6). As the fifth subunit of CSN [47], CSN5 plays an important role in regulating plant growth, development and various abiotic or biotic stress tolerances. It interacts with CRLs to regulate protein ubiquitination and degradation by regulating the ubiquitin/26S proteasome system [33]. It also interacts with CRLs to regulate various plant hormone-related signal transduction pathways, such as auxin, jasmonic acid and salicylic acid signals [26,27,28,29]. In *Arabidopsis*, CSN5 interacts with the ascorbic acid (AsA) biosynthetic enzyme GDP-Man pyrophosphorylase (VTC1) to inhibit the synthesis of AsA, thus negatively regulating the salt stress tolerance of plants [30]. It also interacts with CRLs to assist in the plant response to cold stress through ubiquitination [48]. Cui et al. reported that the silencing of the *CSN5* gene in susceptible grapevine enhanced resistance to powdery mildew [49]. The expression levels of the SA-associated marker genes *PR1*, *PR3* and *PAD4* were higher in the *CSN5*-RNAi plants and lower in the *CSN5*-overexpressing plants than in the WT plants.

Several studies have reported that CSN5 regulates plant resistance to pathogen and pest invasion by activating the JA pathway while inhibiting the SA pathway. Shang et al. [29] reported that tomato CSN4 and CSN5 play critical positive roles in resistance against root-knot nematodes (RKNs), which is positively related to JA content. The downregulation of tomato CSN5 resulted in reduced resistance to *Botrytis cinerea* and *Manduca sexta* larvae, which corresponded with reduced JA content [50]. Zhang et al. [51] reported that CSN5 negatively regulates the resistance of wheat to *Puccinia triticina*, as well as *Puccinia striiformis* f. sp. *tritici*, which is closely related to the SA pathway [28]. In our study, the expression of *StCSN5* was significantly induced by SA, JA and ETH (Figure 7), suggesting that StCSN5 is also involved in the response to phytohormone signaling. The StPAM16-1 protein contains a highly conserved DnaJ domain (Figure 1a) that binds to the heat shock protein for its proper folding, thus playing key roles in reactive oxygen species (ROS) scavenging and signaling [52]. When StPAM16-1 interacts with StCSN5, we hypothesize that the binding of StPAM16 to StCSN5 may assure the correct folding of StCSN5 and enable it to participate in the regulation of resistance pathways such as phytohormone signaling response. Moreover, as StPAM16-1 interacted with StCSN5 and the expression levels of both genes were induced by TA, we speculated that after TA treatment, an increase in the amount of the StCSN5 protein might be needed to degrade the accumulated StPAM16-1 protein to assist in plant defense. However, it is also possible that StPAM16-1 and StCSN5 form a complex to synergistically regulate the host immune response.

Further investigations will focus on the correlation between StPAM16-1 and defensive pathways, including phytohormone, metabolism and R-related genes. Generating *StCSN5*-silenced plants and investigating the biochemical activity of StCSN5 may provide more insights into the function of StPAM16-1-StCSN5 in the host immune response.

## 4. Materials and Methods

### 4.1. Plant Growth and Treatments

The tetraploid potato (*Solanum tuberosum* L.) cultivar Shepody was micropropagated on solid Murashige and Skoog (MS) medium and subcultured every four weeks at 23 °C with a 16 h light–8 h dark photoperiod. Three or four weeks later, potato plants with the same node segments and similar lengths were transplanted into pots containing nutrient soil and vermiculite (*v*/*v* = 3:1) and grown in a greenhouse under the same conditions as those used for tissue-cultured potato plants. Potato plants were watered with tap water once a week and Hogland nutrient solution once a month. *N. benthamiana* plants were grown in the greenhouse at 26 °C with the same photoperiod as that of the potato plants. For TA and hormone treatments, potato plants grown for three weeks were treated with 10 μM TA, 1 mM SA, 50 μM JA or 1 mM ETH and sampled at the indicated time points for further analysis. Leaves treated with sterile water were used as controls. A total of 3 pots (25 cm in diameter) with 5 seedlings per pot were used to replicate each treatment.

### 4.2. Pathogen Cultivation and Inoculation

The *S. scabiei* strain 4.1765, a typical pathogenic isolate from the China General Microbiological Culture Collection Center (CGMCC), was used in this study. *S. scabiei* was cultured on oatmeal agar medium (OMA) plates at 28 °C. Then, the spores were scraped off and dissolved in sterile water to make the spore suspension. The concentration of the spore suspension was adjusted to approximately 1 × 10^7^ CFU/mL using the flat colony counting method. A diluted spore suspension was used to inoculate 3-week-old potato plants.

CS disease assessment was performed as previously described. Tubers > 2 g were selected and washed under running water, and then CS severity was evaluated. The disease index was calculated by the following equation: Disease index = [∑(n × 1 + n × 2 + n × 3 + n × 4 + n × 5)/(N × 5)] × 100. (n = number of tubers corresponding to the numerical grade. N = total number of potato tubers assessed. 5 = high score on the severity of scale). The percentage of tuber area covered was 0. No symptoms of scab; 1. 0–12.5%; 2. 12.6–25%; 3. 26–50%; 4. 51–75%; 5. 76–100%. Control efficacy = (disease index of control − disease index of treated)/disease index of control × 100%. Rate of disease = [number of infected tubers/total number of tubers] × 100× 100%.

### 4.3. Subcellular Localization of StPAM16-1

The coding region of *StPAM16-1* was amplified by PCR and fused with GFP under the control of the CaMV 35S promoter in the plant expression vector pGWB6. Plasmids containing GFP or mitochondrial markers were used as controls. The plasmids were transformed into *Agrobacterium tumefaciens* strain GV3101 competent cells, which were subsequently infiltrated into the leaves of 4-week-old *N. benthamiana* plants. After 48 h of incubation, fluorescent signals were visualized using a confocal laser scanning microscope (Leica TCS SP8, Leica Microsystems, Wetzlar, Germany).

### 4.4. Virus-Induced Gene Silencing

A specific cDNA fragment of *StPAM16-1* was amplified by PCR and inserted into the plant expression vector pgR107. pgR107 and pgR107-StPDS were used as negative and positive controls, respectively. Then, the plasmids were transformed into *A. tumefaciens* strain GV3101 competent cells (containing pJIC SA_Rep), which were subsequently infiltrated into 2-week-old potato leaves [53]. Potato plants were incubated in the dark for 24 h and then transferred to a greenhouse. Potato plants harboring pgR107-StPDS were used as a positive control.

### 4.5. Vector Construction and Potato Transformation

The specific cDNA fragment of *StPAM16-1* was amplified by PCR and inserted into the plant expression vector pANDA35K under the control of the CaMV 35S promoter. Then, the plasmid was transformed into GV3101 competent cells. For potato transformation, the stems of 3-week-old sterile potato plants were cut into 1 cm long pieces and immersed in A. tumefaciens resuspension solution for 10 min. After removing the excess solution, the stems were cultured on MS1 (MS + 4 mg/mL zeatin + 1 mg/mL indole-3-acetic acid) medium in the dark for 2 d. Then, the stems were transferred to MS2 (MS1 + 100 mg/L kanamycin + 200 mg/L cephalosporins) medium for 3–4 weeks to produce callus. The seedlings grown from callus were transplanted into MS3 (MS1 + 100 mg/L kanamycin) medium, and transgenic identification was carried out after the plants developed roots.

### 4.6. RNA Extraction and RT-qPCR Analysis

Total RNA was extracted from various potato tissues using a Plant Total RNA Extraction Kit (Tiangen, Beijing, China) according to the manufacturer’s instructions. The same tissues from 5 plants in one pot were pooled together as one sample for RNA extraction. Three replicates were performed for each tissue sample. Then, cDNA was synthesized from 2 μg of RNA using TransScript^®^ One-Step gDNA Removal and cDNA Synthesis SuperMix (Transgene, Beijing, China). RT-qPCR was performed using SYBR^®^ Green Real-time PCR Master Mix (Toyobo, Osaka, Japan). All RT-qPCR experiments were performed using a CFX96 Touch instrument (Bio-Rad, Hercules, CA, USA) with three technical replicates and biological replicates. The relative expression of genes was calculated by the 2^−∆∆Ct^ method. *StActin* (PGSC0003DMG400027746) was used as the reference gene. The primers used for RT-qPCR are shown in Table S1.

### 4.7. Y2H Assay

The Y2H assay was performed according to the Matchmaker Gold Yeast Two-Hybrid System’s instructions (Clontech, Mountain View, CA, USA). The ORFs of *StPAM16-1* and *StCSN5* were inserted into pGBKT7 and pGADT7, respectively. The plasmids BD-53/AD-T, BD-StPAM16-1/AD, BD/AD-StCSN5, BD/AD and BD-StPAM16-1/AD-StCSN5 were co-transformed into yeast strain AH109 competent cells, which were subsequently cultured on DDO medium (SD medium/-Leu/-Trp) at 28–30 °C for 3–5 days. The yeast cells were subsequently resuspended and cultured in QOD medium (SD medium/-Leu/-Trp/-His/-Ade) supplemented with 40 μg/mL X-α-Gal at 30 °C. Then, the growth of colonies was observed and photographed.

### 4.8. Split Luciferase Complementation Assays

For the firefly luciferase complementation imaging assay [47], the ORF regions of *StPAM16-1* and *StCSN5* were inserted into the plant expression vectors pCAMBIA1300-CLuc and pCAMBIA1300-NLuc, respectively, and subsequently transformed into *Agrobacterium* cell strain GV3101. The Agrobacterium cultures were mixed and co-injected into the leaves of 4-week-old *N. benthamiana* plants. The plants were cultured in the dark for 24 h and then transferred to a greenhouse for 24 h. The leaves were sprayed with 0.5 mM D-luciferin potassium salt solution, and the Luc signals were captured using a plant in vivo imaging system (NightSHADE LB 985, Berthold Technologies, Baden, Germany).

### 4.9. Trypan Blue Staining

Trypan blue staining was performed as described previously [35]. Briefly, potato leaves were placed in a small beaker and washed with distilled water three times. The trypan blue staining solution (0.02% m/v trypan blue; ethanol-phenol-water-83% lactic acid = 2:1:1:1) was added to the beaker and boiled for 5–10 min. Then, 2.5 g/mL chloral hydrate solution was added to the beaker for decolorization, and the leaves were photographed.

### 4.10. Phylogenetic Analysis

PAM16-1 and relative homologous proteins sequences were downloaded from Potato Genomics Resource (http://solanaceae.plantbiology.msu.edu/, accessed on 15 November 2021) and NCBI (http://blast.ncbi.nlm.nih.gov/, accessed on 15 November 2021) databases. Multiple sequence alignment and phylogenetic analysis were performed using DNAMAN and MEGA7.0 software. Phylogenetic tree was constructed using Neighbor-joining method.

### 4.11. Statistical Analysis

Statistical analysis was performed using GraphPad Prism 9.3.0 (GraphPad Software, Boston, MA, USA). Three independent replications were performed for each test, and all values are reported as the mean with standard deviations (SDs). Student’s *t* test (unpaired, two-tailed) was used to test for statistical significance for two groups compared at the 95% significance level (*p* < 0.05) or 99% significance level (*p* < 0.01).

## 5. Conclusions

Potato CS is a soil-borne disease that seriously threatens tuber quality and its market value. To date, an effective approach to controlling CS is lacking. In this study, we identified a gene designated *StPAM16-1* that responds to the phytotoxin TA and demonstrated that StPAM16-1 acted as a negative regulator of CS defense in potato. Importantly, we found that StPAM16-1 localized to the mitochondria and interacted with the COP9 signalosome subunit StCSN5, and the expression of StCSN5 significantly increased in potato leaves after they were treated with TA. These results provide important clues for understanding the functional mechanism of StPAM16-1. The identification of important regulators involved in the interaction between potato and *S. scabiei* could provide candidate genes for the genetic manipulation of plants with increased disease resistance. Based on our results, we propose that *StPAM16-1* can be used as a target gene for gene editing to develop new varieties of potato resistant to CS. Moreover, after the function of StCSN5 was verified, *StCSN5* could also be used as a target gene.

## Figures and Tables

**Table 1:** StPAM16-1-interacting proteins.

Number	Protein Description	NCBI Database Accession No.
1	PREDICTED: COP9 signalosome complex subunit 5b-like protein	XP_006351153.1
2	PREDICTED: anthocyanidin 3-O-glucosyltransferase 2-like protein	XP_006353729.1
3	fructose-bisphosphate aldolase-like protein	NP_001275379.1
4	PREDICTED: uncharacterized protein LOC102596406	XP_006344642.1

## Data Availability

All data generated or analyzed during this study are included in the published article and Supplementary Materials.

## Supplementary Materials

The following supporting information can be downloaded at https://www.mdpi.com/article/10.3390/ijms26031361/s1.
